# Intrauterine growth restriction and congenital malformations: a retrospective epidemiological study

**DOI:** 10.1186/1824-7288-39-23

**Published:** 2013-04-11

**Authors:** Giuseppe Puccio, Mario Giuffré, Maria Piccione, Ettore Piro, Grazia Rinaudo, Giovanni Corsello

**Affiliations:** 1 Dipartimento di Scienze per la Promozione della Salute e Materno Infantile, Università degli Studi di Palermo, Palermo, Italy

**Keywords:** Congenital malformations, SGA, Weight percentile, Ponderal index

## Abstract

**Background:**

Intrauterine growth restriction (IUGR) and small for gestational age (SGA) birth have been considered possible indicators of the presence of malformations. The aim of this study is to evaluate such relationships in a population of newborns, along with other epidemiological and auxological parameters, in particular the ponderal index (PI).

**Methods:**

We analyzed the birth data of 1093 infants, classified according to weight for gestational age as SGA, appropriate for gestational age (AGA) or large for gestational age (LGA). The prevalence of malformations was analyzed in relation to weight percentile at birth and SGA birth, maternal smoking, pregnancy diseases and PI.

**Results:**

Our analysis showed no significant relationship between the prevalence of malformations and SGA birth. Maternal smoking and pregnancy diseases were strongly related to SGA birth, but not to a higher prevalence of malformations. PI, however, had a significant relationship with a higher prevalence of malformations, if analyzed as either a continuous variable or a categorical variable (cutoff: < 2.4).

**Conclusions:**

The association between congenital malformations and birth weight for gestational age seems to be weak. As part of diagnostic screening for malformations in the neonatal period, PI could be considered a better predictor of risk than weight percentile.

## Background

Intrauterine growth restriction (IUGR) can be either symmetric or asymmetric. Symmetric IUGR is characterized by a similar and proportionate reduction in all auxological parameters, including weight, length, and cranial and abdominal circumference. On the other hand, asymmetric IUGR is characterized by a greater reduction in body weight, when compared to length. The diagnosis of IUGR is not always easy or direct; small for gestational age (SGA) birth is usually considered an indirect indicator of IUGR, even if these two concepts are not identical.

It is thought that IUGR is related to the presence of congenital malformations due to at least two possible pathogenetic mechanisms [1];

growth retardation occurs as a result of a congenital malformation

growth retardation and congenital malformations occur as different manifestations of a common, independent etiological factor.

The offspring of multiple pregnancies show an increased incidence of prematurity, a lower birth weight and a higher prevalence of congenital malformations at birth [2].

Lituania et al. found a higher risk of malformations in newborns with IUGR; they suggest that antenatal IUGR, especially when found to occur during the early stages of pregnancy, may be caused by the presence of malformations and chromosomal abnormalities [3]. Khoury et al. demonstrated similar results when they reported that the prevalence of IUGR is higher in newborns with malformations than in normal babies [4].

Dashe et al. analyzed the prevalence of malformations in newborns appropriate for gestational age (AGA), or with symmetric SGA, versus those with asymmetric SGA. They found in their cohort that asymmetric SGA infants were at significantly higher risk of major malformations (14%) compared with AGA and symmetric SGA babies (4% and 3%, respectively, p<0.001). In addition, aneuploidy was more common (p <0.001) in asymmetric SGA newborns (3%), compared with symmetric SGA (1%) and AGA newborns (less than 0.1%) [5].

Other studies have focused on specific types of malformations and chromosomal abnormalities, and on how those specific conditions may be related to IUGR and/or SGA birth. Several case–control studies and case series have analyzed the association between intrauterine growth restriction, low birth weight (less than 1500 grams) or SGA birth, and various types of malformations, such as congenital heart disease (transposition of the great arteries, tetralogy of Fallot, hypoplastic left heart syndrome, etc.) [6], malformations of the urogenital tract (renal hypoplasia, hypospadias) [7-12], the respiratory system [13], and the gastrointestinal tract [14-16].

Particularly interesting are the studies on the relationship between IUGR and chromosomal abnormalities and/or genetic syndromes. Several studies, in fact, report that fetuses with chromosomal disorders, including trisomy 13, 21 and 18, often exhibit IUGR. The same has been shown for Turner syndrome. Aneuploidy and genetic syndromes are predominantly associated with asymmetric IUGR [17,18]. Some rare genetic syndromes, such as Cornelia de Lange and Silver Russell syndromes, have also been found to seriously affect intrauterine growth.

The aim of this study is to evaluate, in a population of newborns, the possible relationship between the presence of major congenital malformations and various epidemiological and auxological parameters, in particular low birth weight in relation to gestational age (SGA birth) and ponderal index (PI) as a marker of asymmetric intrauterine growth.

## Methods

In this retrospective study, we analyzed the birth data of all inborn babies admitted to the Neonatal Unit of Azienda Ospedaliera Universitaria Policlinico “Paolo Giaccone” in Palermo, from January 1^st^ 2011 to March 31^st^ 2012. A total of 1093 infants were enrolled, including 34 twins (3.1%). Newborns were classified according to weight for gestational age (GA) in the following categories:using the percentile charts of the Italian Neonatal Study (INeS) 2011 [19], and in particular the computational “smoothing” procedure available at the INeS website (http://www.inescharts.com/index.aspx), based on standard deviation, that assigns an exact percentile value to each newborn.

✓ SGA: weight for GA below the 10^th^ percentile;

✓ AGA: weight for GA between the 10^th^ and 90^th^ percentiles;

✓ LGA (large for GA): weight for GA above the 90^th^ percentile

The PI was computed as follows:

$$\mathrm{PI}=100\times \mathrm{Bodyweight}\left(g\right)/\mathrm{lengt}h^{3}\left(\mathrm{cm}\right)$$

The study population was also divided into two categories according to the presence or absence of congenital malformations. Only major malformations were considered.

Statistical analysis was performed by the open software R [20].

The best cutoff for PI was determined by an ROC curve analysis, using the R package ROCR [21].

## Results

In total, 1091 infants were evaluated (two were excluded because their GA was unknown); 877 (80.38%) were AGA, 76 (6.97%) SGA and 138 (12.65%) LGA.

The GA range was 151–299 days, including preterm newborns (114, 10.45%), term newborns (905, 82.95%) and post-term newborns (72, 6.60%).

The total prevalence of malformations was 4.85% (53 cases). Table 1 summarizes the malformations in our population.

**Table 1:** Malformations observed in our sample population

**Malformations**	**Cases**
Cardiac	14
Multiple	11
Renal	6
Abdominal	5
Down syndrome	4
Facial	3
Cutaneus	2
Genital	2
Ocular	2
Anal	1
Encephalic	1
Osteoarticular	1
Vascolar	1

The prevalence of malformations was 5.1% in SGA, 4.8% in AGA and 5.3% in LGA newborns. These small differences were not statistically significant (p = 0.97).

**Figure 1:**
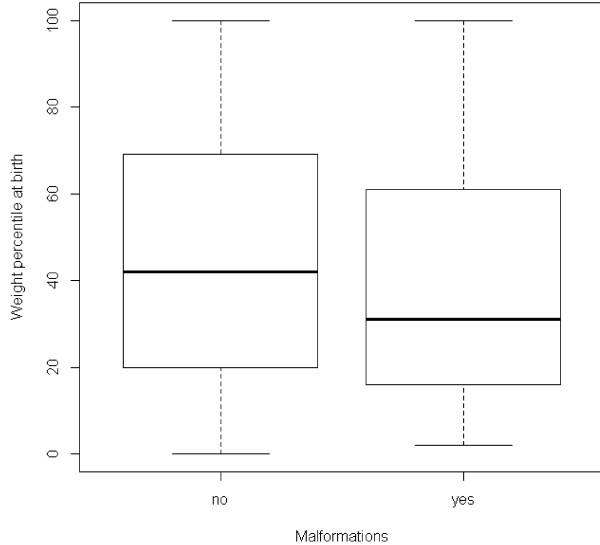
**Weight percentile distribution according to the presence of malformations.** Wilcoxon test, p = 0.078.

Analyzing the distribution of exact weight percentile values according to the presence of malformations, we found that the values were slightly lower in the malformation group (Figure 1). The difference, however, did not reach statistical significance (p = 0.078).

In addition to the presence of malformations, we considered other obstetric factors that can affect birth weight, such as diseases and smoking during pregnancy, to assess their role in determining a condition of IUGR, and their possible relationship with the presence of malformations.

Smoking during pregnancy, defined as the average consumption of five or more cigarettes per day, was present in 11.89% of pregnancies. The prevalence of SGA births was significantly higher in pregnancies in which the mother smoked (22.3%, compared to 11.3% in the non-smoking group, p = 0.0019, chi-square test). Smoking during pregnancy, however, was not associated with the presence of malformations; the prevalence of malformations was similar in infants born to smoking and non-smoking mothers (5.4% vs. 4.8%, p = 0.76).

Diseases in pregnancy (or pregnancy-related) were present in 32.11% of the population, as summarized in Table 2.

**Table 2:** Diseases observed in pregnancy or pregnancy related

**Disease**	**Cases**
Diabetes and gestational diabetes	71
Previous abortion/preterm delivery	54
Recto-vaginal swab positive for GBS	34
Hematological disease	33
Gestosis	28
Thyroid diseases	28
TORCH infection	24
Urinary infection	17
HBV/HCV/HIV infection	8
Uterine fibromioma	6
Choriamnionitis	1
Other	17

The prevalence of SGA births was significantly higher in mothers with disease (18.0% versus 10.1% in the non-disease group, p = 0.00092, chi-square test), but we found no relationship between maternal disease and malformations, which were present in 3.7% of pregnancies with diseases and in 5.4% of pregnancies without diseases (not statistically significant, p = 0.23).

As SGA babies did not seem to have a higher prevalence of malformations in our sample population, we tried to adopt a stricter definition of IUGR, considering only newborns whose weight percentile was lower than 3 (“extreme SGA”). There were 41 such newborns in our population (3.76%).

Again, we could not observe any differences in the prevalence of malformations in the two groups (4.9% in “extreme SGA”, 4.9% in the others, p = 0.99).

Finally, we considered the PI, an indicator of asymmetric intrauterine growth, introduced for the first time by Rohrer in 1921. In our population, the PI mean value was 2.67 ± 0.25, median 2.67, range 1.77 - 3.60.

**Figure 2:**
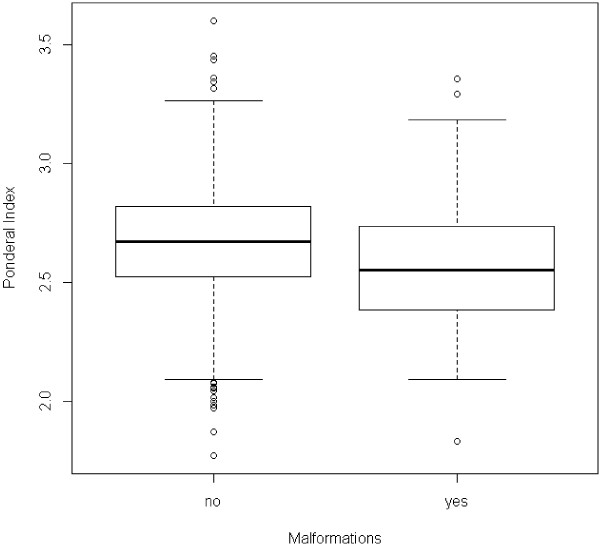
**Ponderal Index distribution according to the presence of malformations.** Wilcoxon test, p = 0.006.

Figure 2 shows the distribution of PI depending on the presence or absence of malformations. The PI tended to be lower in newborns with malformations, and the difference was statistically significant (p = 0.006, Wilcoxon test).

**Figure 3:**
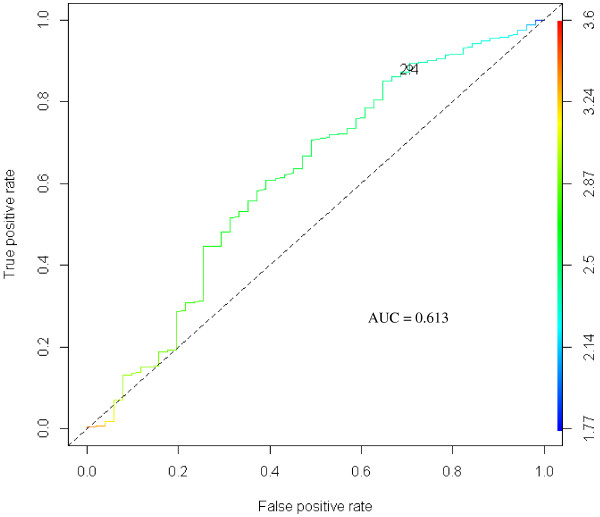
**ROC curve of PI as a predictor for malformations.** A ROC curve of the trade-off between true positive rate and false positive rate measures across the range of all cutoffs. The curve is parametrized with the cutoff.

**Figure 4:**
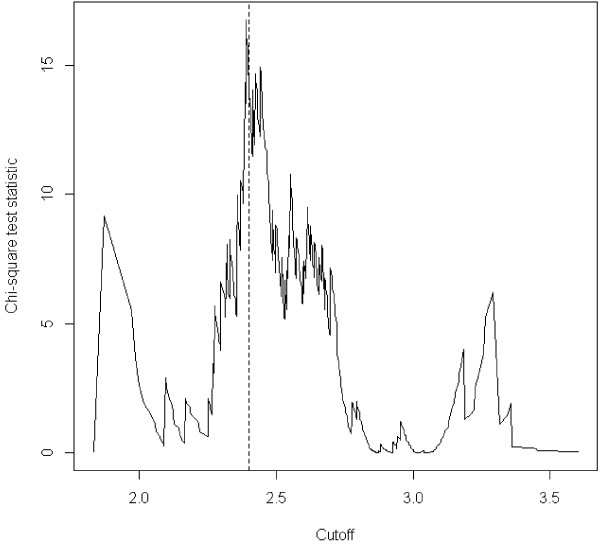
**Chi square values across the range of cutoffs for PI as a predictor for malformations.** The vertical line corresponds to the chosen cutoff of 2.4.

To test PI as a categorical variable, we performed an ROC curve analysis using PI as a predictor of malformations. Considering both an accuracy curve and a chi square curve, a value of 2.4 was chosen as the best cutoff in predicting the presence of malformations (Figures 3, 4).

We considered as “asymmetric” newborns those with a PI <2.4. In our population, 12.55% of newborns were asymmetric. The prevalence of malformations was significantly higher in the group of asymmetric newborns (11% vs. 3.8%, p = 0.0002, chi-square test).

**Figure 5:**
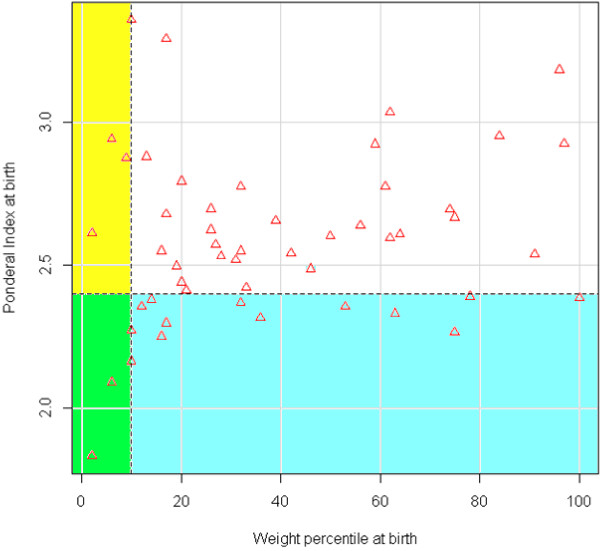
**Weight percentile at birth and Ponderal Index at birth in babies with congenital malformations.** Horizontal dashed line: PI cutoff (2.4); Vertical dashed line: Weight percentile cutoff (10); Yellow and green rectangles: cases with weight percentile lower than 10; Blue and green rectangles: cases with PI lower than 2.4; Green rectangle: cases with weight percentile lower than 10 and PI lower than 2.4 (only babies with congenital malformations).

Figure 5 summarizes the relationship between the presence of malformations, weight percentile and PI. Only a very small number of babies with malformations were both SGA and asymmetric (green rectangle), or simply SGA (yellow rectangle), while many more presented only with asymmetry of growth (blue rectangle). The majority of newborns with malformations, however, were neither SGA nor asymmetric.

**Figure 6:**
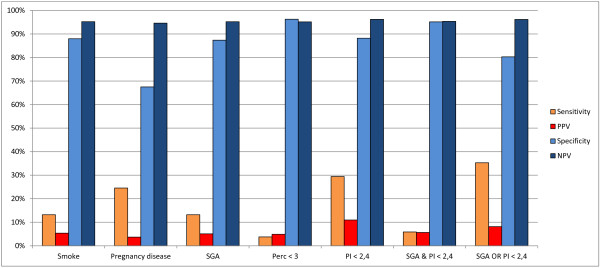
**Sensitivity, Specificity, PPV, NPV of different parameters considered as predictors of malformations.** PPV = Positive Predictive Value; NPV = Negative Predictive Value; CI = Confidence Interval; Perc = weight percentile at birth; PI = Ponderal Index at birth; SGA = Small for Gestational Age newborn.

We also expressed the relationship between the above variables and malformations as the sensitivity, specificity, positive predictive value (PPV) and negative predictive value (NPV) of each variable, considered as an indicator of the presence of malformations. Table 3 and Figure 6 summarize the results, with 95% confidence intervals and p-values (chi-square test).

**Table 3:** Sensitivity, Specificity, PPV, NPV of different parameters considered as predictors of malformations

	**Sensitivity**	**95%CI**	**Specificity**	**95%CI**	**PPV**	**95%CI**	**NPV**	**95%CI**	**p value**
**Smoke**	13.21%	5.48- 25.34	88.00%	86.05 - 90.07	5.38%	2.19 - 10.78	95.22%	93.68 - 96.48	0.76
**Pregnancy disease**	24.53%	13.75-38.28	67.50%	64.56 - 70.34	3.70%	1.99 - 6.25	94.61%	92.73 - 96.12	0.23
**SGA**	13.21%	5.48 - 25.34	87.38%	85.20 - 89.34	5.07%	2.06 - 10.17	95.17%	93.61 - 96.44	0.97
**Perc < 3**	3.77%	0.46 - 12.98	96.24%	94.90 - 97.31	4.88%	0.60 - 16.53	95.14%	93.66 - 96.36	0.99
**PI < 2.4**	29.41%	17.49- 43.83	88.19%	86.06 - 90.10	10.95%	6.26 - 17.41	96.20%	94.76 - 97.32	0.00019
**SGA & PI < 2.4**	5.88%	1.23 - 16.24	95.15%	93.66 - 96.38	5.66%	1.18 - 15.66	95.33%	93.86 - 96.54	0.74
**SGA OR PI < 2.4**	35.29%	22.43- 49.93	80.31%	77.75 - 82.60	8.14%	4.90 - 12.57	96.17%	94.66 - 97.35	0.007

*PPV* = Positive predictive value; *NPV* = Negative predictive value; *CI* = Confidence interval; *Perc* = weight percentile at birth; *PI* = Ponderal index at birth; *SGA* = Small for gestational age newborn.

Smoking during pregnancy and diseases in pregnancy had no significant association with malformations and low sensitivity-specificity values. More interestingly, the condition of SGA behaved approximately in the same way, and was not a good predictor of malformations. Similar results were also observed for the condition of extreme SGA (below the 3^rd^ percentile), where, predictably, specificity was higher, but sensitivity was very low.

When we considered PI, however, categorized at the cutoff of 2.4, the statistical association was highly significant (p = 0.00019), and we observed discrete values of sensitivity (29.41%) without losing too much in specificity (88.19%). PPV and NPV also confirmed this trend.

Finally, we evaluated the predictive value of the association of the SGA condition with low PI, considering both their intersection and their union (AND and OR). Significant values were obtained only for the union (OR), but using the union of both variables did not improve the results observed for PI alone; sensitivity, predictably, was a little higher, but at the expense of specificity, and the statistical association became weaker (p = 0.007).

The analysis in terms of sensitivity and specificity confirmed what could be inferred from the initial analysis: only PI seemed to have some predictive value for the presence of malformations. The other variables considered, including the percentile of weight, were not associated with malformations, or the association was probably too weak to be detected in a limited series of cases. In other words, newborns with low PI seemed to be at higher risk of having malformations, while newborns with low weight percentile at birth (lower than 10, or even lower than 3) apparently had no increased risk.

**Figure 7:**
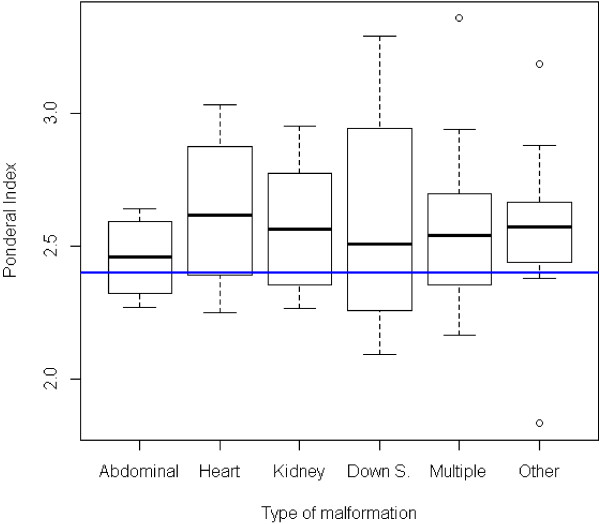
**Ponderal Index distribution according to the type of malformation.** Horizontal blue line: PI cutoff (2.4); Kruskal Wallis test, p = 0.93.

Finally, Figure 7 shows the distribution of PI in the various classes of malformations. While the differences were absolutely non-significant as a whole, we could observe that lower PI values were more frequent in abdominal malformations and in Down syndrome, and less evident in renal or multiple malformations, while cardiac malformations and other forms seemed not to be associated with a lower PI.

## Discussion

SGA newborns are certainly a group whose nature is very heterogeneous, including some absolutely normal babies, “smaller” than the rest of the population for constitutional reasons, and newborns affected by different kinds of disease. Typically, “symmetric” SGA newborns, if pathological, are considered to be affected mainly by genetic, chromosomal and infectious diseases or any other condition affecting the growth of the fetus in the early stages of gestation, while “asymmetric” SGAs are often explained by placental pathologies in late pregnancy.

As congenital malformations, or at least some of them, can interfere with normal fetal growth, it is generally assumed that SGA newborns may have a higher prevalence of malformations.

In our series, we observed that newborns with birth defects had a weight percentile slightly lower than those in the general population (Figure 1), but the difference was not significant. The effect could probably be real but weak, considering the relatively low number of newborns with birth defects in our sample population.

The prevalence of malformations in the SGA group, however, was definitely comparable to its prevalence in the AGA and LGA groups. Very likely, the small difference in the weight percentile distribution that could be observed between babies with and without malformations is not strong enough to cause a significantly different rate of malformations in the SGA group.

We found, however, that PI was a more sensitive marker of malformations. As can be seen in Figure 5, the number of malformed newborns having a low PI at birth was greater than the number of those having a weight percentile lower than 10. A possible explanation for that is that some malformations may predominantly affect weight during fetal life, expressing themselves more as asymmetric growth rather than as a significant reduction in the weight percentile.

Other epidemiological parameters that we considered, such as smoking in pregnancy and disease in pregnancy, can certainly cause some impairment of fetal growth, but, as might be expected, had no special association with the risk of malformations.

## Conclusions

In summary, the association between congenital malformations and birth weight seems to be real, but weak, and it is apparent more as an asymmetry of weight versus length than as an absolute reduction in the weight percentile.

Particular attention should be given to the detection of malformations at birth, especially those not clinically obvious. Screening procedures may include ultrasonographic evaluation in babies who have a higher risk of malformations.

According to our data, a low weight percentile at birth may not be a reliable indicator of such a higher risk. On the contrary, a low PI, as an expression of asymmetric growth, could be considered a more valuable predictor of risk and therefore could be used to select a specific subgroup of newborns which should receive further diagnostic attention.

## Competing interests

The authors declare that they have no competing interests.

## Authors’ contributions

GP made substantial contributions to conception and design of the study, and to analysis and interpretation of data, was involved in drafting the manuscript and revising it, and gave final approval of the version to be published. MG made substantial contributions to acquisition of data, was involved in drafting the manuscript, and gave final approval of the version to be published. MP made substantial contributions to acquisition of data, was involved in drafting the manuscript, and gave final approval of the version to be published. EP made substantial contributions to acquisition of data, was involved in drafting the manuscript, and gave final approval of the version to be published. GR made substantial contributions to the design of the study, to acquisition of data and to analysis and interpretation of data, was involved in drafting the manuscript, and gave final approval of the version to be published. GC made substantial contributions to conception and design of the study, was involved in drafting the manuscript and revising it critically for important intellectual content, and gave final approval of the version to be published. All authors read and approved the final manuscript.
